# Reform of the London College of Surgeons

**Published:** 1840-01

**Authors:** 


### REFORM OF THE LONDON COLLEGE OF SURGEONS.

Since the preceding observations were in type, an excellent letter has appeared from Mr. Key (in the *Medical Gazette* of Dec. 6), on the necessity of reform in the constitution of the London College of Surgeons. Did our space permit, we should willingly transcribe the whole of this communication, which, we doubt not, will produce an impression commensurate with the high character, distinguished abilities, and elevated professional station of the author. We can only find room, however, for a small portion of it; but the extracts will prove, by the highest possible evidence, the justness of our preceding remark, that "this department, also, stands in need of some reform." Mr. Key's views, it will be seen, are much less comprehensive than ours; but we gladly hail the accession to our cause of so able an advocate, although he may be, as yet, prepared to go only a part of the way with us. We cannot, however, but hope, from the liberal and clear views displayed in Mr. Key's letter, that a further consideration of the subject will lead him to regard the reform of the College of Surgeons as only a part of the general reform, which we conceive to be indispensable for the honour and welfare of the profession. If the College of Surgeons and College of Physicians would only condescend to read the signs of the times, and deign to be wise, even now in this their day; instead of opposing, they would cooperate with all honest reformers in bringing about the contemplated and inevitable changes: otherwise, they will probably lose even the scientific and departmental influence which would naturally attach to them, and the possession of which, as sections of one body representing the whole profession, would not be incompatible with the most thorough reform.

.... "Can it be said that the College is popular with the profession? The members, it is true, seek to obtain its diploma, because without it some public appointments cannot be held; and, till lately, no other chartered body has had the power of conducting surgical examination. The degree of popularity that it does enjoy is owing to the individual weight of one or two examiners, and not to the whole as a body. Is it popular with the country? The answer is, that a petition to parliament for such powers as had been granted to the Apothecaries' Company was made, and refused; and the profession saw in silence the failure of the application. On occasions when the proceedings of the College have been assailed without regard to truth, and misrepresented and distorted to serve party purposes, not a voice has been raised in its defence. The Royal College of Surgeons is sometimes pledged as a toast at a public dinner, but more as a matter of form than of feeling; and then something cold is said by way of compliment, and something as cold replied by a member of council, as the expression of thanks for the honour conferred; its prosperity excites in its members no feeling of pride, as its degradation would none of sympathy.

"Why is the College, with its talent, respectability, and great resources, regarded by the profession with so cold, so jealous an eye? It is because the members have no community of feeling with the College. The student, when he becomes a member on receiving his diploma, and leaves the door of the building, feels that he has no closer connexion with it than before his name was enrolled. He asks himself, and asks in vain, what advantage he derives from the fee that he has paid, and from the severe self-imposed examination that he has undergone. His hopes and fears, once centered in the College, end with his examination. In truth, he can hardly be said to have any connexion with the College. If he chance to live in London, he may hear, if he please, an occasional lecture, and may have access to the library—advantages that his former school of medicine still continue to afford him. It is no *Alma Mater* to him. He may long for its honours, but they are beyond his grasp; he feels that no industry, no exertion of talent, can place them within his reach. As he advances in life, he sees his former fellow-student, possessing half his industry, talent, and knowledge, called to the council of the College, from which he is excluded, and raised to the rank of examiner, which he never can attain. He has no voice in the election of the council, much less in its measures; he feels himself a cypher, if not an alien, and that he is so regarded by the College. Surely there is nothing in this system to attract, but everything to alienate from the College, the affection of its members.....

...."By what rule a member is elected to the council I do not exactly know. To be a London surgeon, and not to be a general practitioner, seem to be two of the necessary qualifications. He need not be a surgeon to an hospital, for persons are selected who are known only as private practitioners. He need not be a surgeon of high professional reputation, for some are of the council whose scientific attainments will not bear a close scrutiny. There is a conventional rule, I conclude, not well defined, but sufficiently understood, to guide the election of the members of the council. Thus entrenched, the governing body of the College knows that it is not popular, and endeavours to atone by legislation for the defects of its system. The exclusiveness that prevails mars their best intentions, leading to all kinds of evils, both to their own body and to the profession; among others, to measures of doubtful policy, in order to conciliate popular feeling. The two last alterations in the laws of education will illustrate what I mean. One is, the abolition of apprenticeships, and the limitation of the period of study to four years. Those who brought forward such a law must surely have forgotten how much, or rather how little, they themselves knew at the end of four years; that the increased severity of their examination requires rather a prolonged than a curtailed period of study: they might also bear in mind, that it takes as much time to make a good surgeon as it does to make a good carpenter. But for this error there is an excuse, as it would seem to be only a show of liberality; for the law is wholly inoperative, while the hall retains its period of apprenticeship. The other is, the committing education wholly to provincial schools. It cannot be said that either of these measures is calculated to raise the standard of education; but they are both popular, and so far answer their purpose. One is probably intended to conciliate the good will of students; the other, that of provincial surgeons; and the end is thought to be gained. The policy is short-sighted. The council cannot suppose that the obvious inference from their own law is overlooked by provincial surgeons; namely, that if their schools are qualified to conduct the whole of education, and to be placed on an equality with the London schools, the leading provincial surgeons ought to be placed on a footing with the London surgeons, in respect to eligibility to the council: and why should they not? Besides, all these small acts of legislation, as expediency may drive, cannot have a lasting good effect: they have the semblance of liberality, but not the substance. The surgeon and the student still remember that they are excluded from the honours of the College, and, forgetting the boon, think only of what is withheld from them. To meet the coming difficulty, the College has three modes of proceeding....

..."They may apply at once to parliament to strengthen their powers, and to make the examination, which at present is voluntary on the part of the student, compulsory. ..... Another line of policy is, to wait the tide of events, and the demolition of the frail fabric of the university [of London.] ..... A third course, by which alone its strength can be permanently increased, is to rest its claim for support on the attachment and esteem of its own members. This can only be done by allowing to each member a vote in election of the members of council. Such a measure would make the College what it has not yet been—a representation of the whole body of surgeons, instead of being, as it now is, a small self-elected section, taking into its own hands the management of its internal concerns, and the legislation for the whole of the profession. The council and the members would form one powerful body, acting in unison, for the common good; the feelings of the members, now diverted from the college, would be concentrated towards it. They would join in measures for its advancement, and in defending it against attacks, which, if made, would then be few and feeble; honorable ambition would be encouraged; and those who work for the profession would replace those who do nothing for science. The libeller would be silenced, and the discontented would be satisfied. Thus strengthened, the College might apply with confidence to parliament for any reasonable extension of powers. The council would then embody the surgical talent of the country in a most popular form. . . . . .

. . . . "Objections that will be urged against such a measure are such as are made against popular elections in general. An election of members of council by a learned body cannot be termed popular, in the objectionable sense of the term. A popular election is defective, from the passions or interests of the electors giving them an improper bias, or from ignorance of the qualification necessary for a member of the council. The interest and feelings of the member would be identified with the College, and no improper motive would be likely to interfere with a sound exercise of their privilege. . . . . .

. . . . "It may be said, that the leading members of the profession would not submit to the annoyance of a canvass and a popular election, and would not come forward as candidates; while men of doubtful pretension, by force of intrigue and noise, might be preferred. It might be so ordered, that the names of candidates should be laid before the profession for the period subsequent to the last election, in order to give time for the relative qualification of each to be ascertained. I am far from intending to lower the standard of qualification for a member of council; on the contrary, my wish is to raise it; nor am I at all desirous of depreciating the character of the hospital surgeon, as I cannot be insensible to the opportunities that surgeons long connected with hospitals possess, of cultivating the science and practical part of the profession, and of qualifying themselves in a superior manner, for the discharge of the duties of an examiner. If surgeons of hospitals do their duty towards the profession, by improving its science and raising its character, the profession in return will not fail to elect them, as the fittest persons, to the council of the College. But this fitness, I say, let the members determine.

"Other advantages to be expected from such a change, are not few nor unimportant. The present want of exhibitions will probably be supplied; a motive for enriching the funds of the College, for such an excellent purpose, will then exist, and generous individuals will be prevailed upon so to apply their redundant means. It is probable that it will also have a good effect on the literature as well as the tone of the profession. Crude works, hasty opinions, ill-digested theories, and imperfectly tested modes of practice, will more rarely issue from the press, as they will fail to produce that lasting good impression, which will then become more the object of active and intelligent surgeons.

"A change of measures in any society usually inflicts injury on some; and a change in the mode of election of the council will not be without its consequences to those who, like myself, look forward, in the present course of events, to become members of that body. The council itself will not suffer by the change, and the members generally will benefit by it. The expectants, under the present system of election, will be the only sufferers."

